# Orbital Topology of Chiral Crystals for Orbitronics

**DOI:** 10.1002/adma.202418040

**Published:** 2025-05-02

**Authors:** Kenta Hagiwara, Ying‐Jiun Chen, Dongwook Go, Xin Liang Tan, Sergii Grytsiuk, Kui‐Hon Ou Yang, Guo‐Jiun Shu, Jing Chien, Yi‐Hsin Shen, Xiang‐Lin Huang, Iulia Cojocariu, Vitaliy Feyer, Minn‐Tsong Lin, Stefan Blügel, Claus Michael Schneider, Yuriy Mokrousov, Christian Tusche

**Affiliations:** ^1^ Peter Grünberg Institut (PGI‐6) Forschungszentrum Jülich 52425 Jülich Germany; ^2^ Faculty of Physics University of Duisburg‐Essen 47057 Duisburg Germany; ^3^ Ernst Ruska‐Centre for Microscopy and Spectroscopy with Electrons Forschungszentrum Jülich 52425 Jülich Germany; ^4^ Peter Grünberg Institut (PGI‐1) Forschungszentrum Jülich and JARA 52425 Jülich Germany; ^5^ Institute of Physics Johannes Gutenberg University Mainz 55099 Mainz Germany; ^6^ Department of Physics National Taiwan University Taipei 10617 Taiwan; ^7^ Department of Materials and Mineral Resources Engineering National Taipei University of Technology Taipei 10608 Taiwan; ^8^ Present address: Physics Department University of Trieste Trieste 34127 Italy; ^9^ Institute of Atomic and Molecular Sciences Academia Sinica Taipei 10617 Taiwan; ^10^ Research Center for Applied Sciences Academia Sinica Taipei 11529 Taiwan; ^11^ Department of Physics University of California Davis Davis CA 95616 USA; ^12^ Present address: Department of Applied Physics, The University of Tokyo Tokyo 113‐8656 Japan; ^13^ Present address: Elettra‐Sincrotrone Trieste S.C.p.A Basovizza S.S. 14, Km 163.5 Trieste 34149 Italy; ^14^ Institute of Mineral Resources Engineering National Taipei University of Technology Taipei 10608 Taiwan

**Keywords:** chirality, circular dichroism, fermi arc, momentum microscopy, orbitronics, orbital angular momentum, orbital topology

## Abstract

Chirality is ubiquitous in nature and manifests in a wide range of phenomena including chemical reactions, biological processes, and quantum transport of electrons. In quantum materials, the chirality of fermions, given by the relative directions between the electron spin and momentum, is connected to the band topology of electronic states. This study shows that in structurally chiral materials like CoSi, the orbital angular momentum (OAM) serves as the main driver of a nontrivial band topology in this new class of unconventional topological semimetals, even when spin‐orbit coupling is negligible. A nontrivial orbital‐momentum locking of multifold chiral fermions in the bulk leads to a pronounced OAM texture of the helicoid Fermi arcs at the surface. The study highlights the pivotal role of the orbital degree of freedom for the chirality and topology of electron states, in general, and paves the way towards the application of topological chiral semimetals in orbitronic devices.

## Graphical abstract

Chirality is ubiquitous in nature and constitutional for life. In chiral materials the intrinsic handedness, defined by their crystal structure, becomes a main driver for a novel electronic topology that is based on the orbital angular momentum rather than the electron spin. The pivotal role of the orbital angular momentum makes chiral materials an ideal candidate for orbitronics applications.

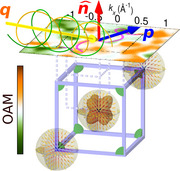

## 1 Introduction

Chirality is ubiquitous in nature and manifested in a wide range of materials and phenomena, from the molecular biology of all organisms to the physical principles governing particle physics. Chiral structures come in pairs known as enantiomers, which are mirror images of each other and cannot be superimposed by rotation and translation. Opposite enantiomers may have completely different chemical/physical properties and respond differently to external stimuli. While many biomolecules that are essential to life exist as single enantiomers, utilizing enantiomeric pairs of quantum materials offers exciting opportunities for tailoring materials with specific functionality. Chiral materials are fascinating due to their representation of the ultimate form of broken symmetry, which is distinct from other forms of symmetry breaking. Recent advancements highlight the potential of chiral materials in optoelectronics and spintronics, demonstrating applications like chiral‐induced spin selectivity,^[^ ^1^ ^]^ circularly polarized beamsplitting,^[^ ^2^ ^]^ and topological transport properties.^[^ ^3^ ^]^

The notion of chirality is also manifested in the low‐energy excitation of electrons in a material. Topological Weyl semimetals host the analog of relativistic particles with a specific chirality (defined as the relative directions between the spin and momentum) in high energy physics, so‐called Weyl fermions.^[^ ^4^, ^5^ ^]^ The chirality of fermions is connected to the topology in the electronic structure through one of the topological numbers, the Chern number *C*. A non‐zero Chern number leads to the formation of a non‐trivial Fermi arc at the surface by connecting a pair of Weyl points with opposite chirality, as the hallmark of a Weyl semimetal.

Generally, symmetry is decisive for the conditions of band crossings and their topological properties in a material. As outlined in **Table** **1**, there is a tendency that as more crystal symmetries are broken, i.e., the symmetries of the respective crystal structure are lower, a higher Chern number *C* can be formed. In non‐magnetic materials, preserved space‐inversion symmetry can lead to a Dirac semimetal with *C* = 0. Broken space‐inversion symmetry can induce chirality in the electronic structure and give rise to a Weyl semimetal with *C* = ±1 despite a non‐chiral crystal structure. Chiral‐structured crystals have no space‐inversion, mirror as well as other roto‐inversion symmetries, and can behave more complex with *C* = ±2 or *C* = ±4.^[^ ^6^ ^]^ Moreover, chiral topological semimetals with *C* = ±2 and *C* = ±4 belong to a “unconventional” class, where unconventional fermions emerge which have no analogs in high‐energy physics.^[^ ^7^ ^]^ These low‐energy excitations are denoted by multifold chiral fermions.^[^ ^3^ ^]^ Despite the lack of inversion, mirror reflection, and roto‐inversion symmetries of chiral crystals, stable formation of multifold chiral fermions still require symmetries such as screw rotation.^[^ ^8^ ^]^ Thus, the chiral crystal should be considered as a distinct class with unique symmetry properties.

**Table 1:** Overview of topological semimetals depending on the symmetry of crystal structures.

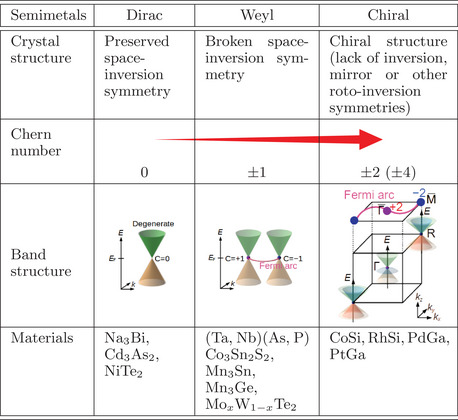

As schematically illustrated in Table 1 for the CoSi‐family topological chiral semimetal, high Chern numbers *C* = ±2 of multifold chiral fermions are found at the Γ and R points. As they are “maximally separated” in the momentum space, exceptionally long Fermi arcs exist at the surface, distinct from conventional Weyl semimetals. Remarkably, the emergence of the multifold chiral fermion in chiral crystals does not require spin‐orbit coupling (SOC), but instead is driven by the structural chirality. The additional consideration of SOC, albeit small, leads to an even larger *C* = ±4, and spin‐split Fermi arcs.^[^ ^9^, ^10^, ^11^ ^]^

In this work, we unveil the nature of the interplay in the topological chiral semimetal CoSi by means of advanced momentum microscopy, which can measure both bulk and surface electronic states with high sensitivity. We utilize the circular dichroism (CD), which serves as a probe for the orbital degree of freedom of the electronic states.^[^ ^12^, ^13^, ^14^, ^15^, ^16^, ^17^ ^]^ In particular, pronounced CD patterns have been reported for multifold chiral Fermions that exist in the bulk electronic structure of topological chiral materials.^[^ ^17^, ^18^ ^]^ While previous work has focussed on the CD and orbital angular momentum (OAM) of the bulk electronic structure, our results link the distinct chirality of the multifold Fermions in CoSi to the OAM texture of the topological helicoid Fermi arcs that emerge on the surface of CoSi. In particular, the OAM texture undergoes a sign change between chiral crystals of opposite handedness. The nontrivial topology of the multifold chiral fermions originates from the orbital chirality in the bulk, and is evidenced by a pronounced OAM polarization of the topological helicoid Fermi arcs. Our first‐principles calculations confirm that the OAM serves as the key link among the crystal chirality, electronic chirality, and topology.

## 2 The Role of the OAM in the Interplay Among Crystal Chirality, Electronic Chirality and Topology

**Figure 1:**
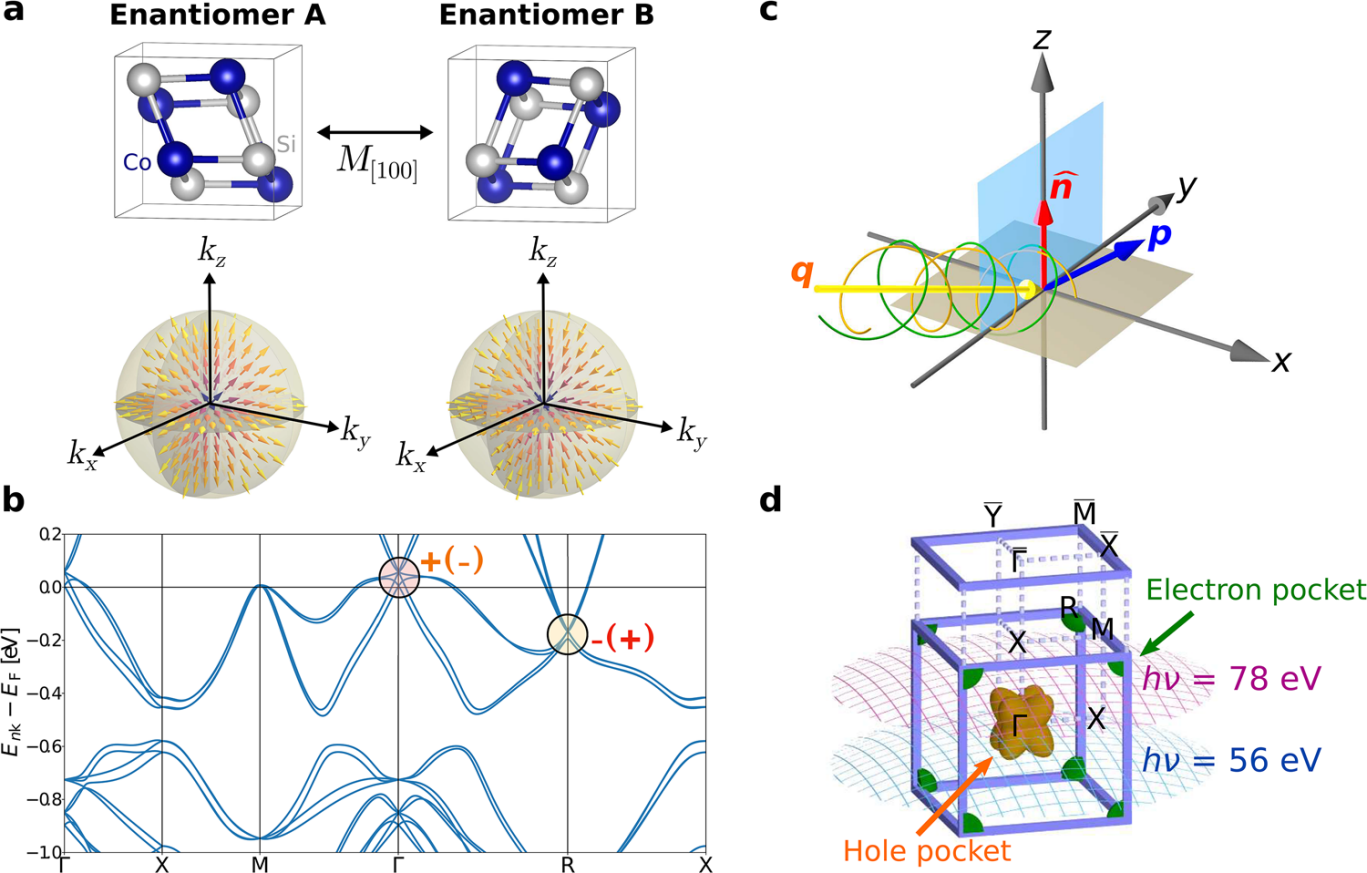
Topological chiral semimetal CoSi and schematics of the experiment. a) The crystal structures of enantiomers A (left) and B (right) are related by the mirror reflection along [100] axis. Below, the **k**‐space OAM texture of a multifold chiral fermion is schematically illustrated. For enantiomers A and B, the orbital chirality **L** · **k** are the opposite. b) Band structure of CoSi. Bulk Γ and *R* points with high Chern numbers are indicated by circles. These charges have opposite signs for enantiomers A and B due to the opposite orbital chirality. c) Experimental photoemission geometry. The photon incidence vector (**q**) lies in the *yz* plane at an angle of 65° with respect to the sample surface normal ($\hat{n}$) and is aligned along the $\bar{Y}-\bar{\Gamma }-\bar{Y}$ direction of the surface BZ. Emitted photoelectrons (**p**) are collected in the full solid angle above the sample surface (*xy*‐plane) by the momentum microscope. d) Schematic Fermi surface of CoSi in the bulk BZ with corresponding spherical sections measured at different photon energies.

The crystal structure of CoSi, which is one of the prototypical chiral topological semimetals, is shown in Figure 1a. The two enantiomer pairs A and B are related via a mirror reflection with respect to the [100] axis. Below, we show the schematics of the OAM textures in **k**‐space of a multifold chiral fermion, where the arrows represent the OAM. The radial and monopole‐like OAM texture has been previously predicted.^[^ ^19^ ^]^ The band structure of bulk CoSi is shown in Figure 1b. The multifold chiral fermions are found at Γ and R, which are highlighted in circles.

Since three orbitals are sufficient to effectively describe the multifold chiral fermion at Γ, which originate from Co *d* orbitals (Figure S8, Supporting Information), the low‐energy Hamiltonian can be written in terms of the *l*_eff_ = 1 OAM operator **L**,

(1) $$H_{k}=\chi v_{F}L\cdot k$$

where **k** is the crystal momentum, χ = ±1 is a parameter determined by the crystal chirality, and *v*_F_ is the Fermi velocity assumed to be constant. Here, **L** can be regarded as an axial vector proportional to the *physical* OAM of Co *d* electrons, whose algebraic properties are isomorphic to that of *p* electrons. The eigenstates are characterized by the orbital quantum number along **k**; $L\cdot \hat{k}|u_{mk}\rangle =m\hbar |u_{mk}\rangle$ (*m* = −1, 0, +1). That is, the OAM exhibits monopole‐like distribution in **k**‐space, as schematically illustrated in Figure 1a. We emphasize that *m* characterizes the OAM chirality, the relative orientation between **L** and **k**. The energy eigenvalues are given by $E_{mk}=\chi v_{F}m\hbar \left|k\right|$. Our first‐principles calculations clearly demonstrate that the OAM evaluated within the atom‐centered approximation exhibits **k**‐space chirality near Γ. The results also show that the OAM chirality is dependent on the crystal chirality (Supporting Information).

The Berry curvature for the eigenstate *u*_*m* **k**_ is given by

(2) Ωmk=−Im⟨∂kumk|×|∂kumk⟩=−mk^|k|2=−⟨umk|L|umk⟩ℏ|k|2

and the Chern number is

(3) $$\begin{array}{ccc} C_{m} & = & -\frac{1}{2\pi }\int dS\cdot \Omega_{mk}=2m=-2,\;0,\;+2 \end{array}$$

This explains the relation between the OAM, Berry curavture, and topology. Meanwhile, the multifold chiral fermion at R is double‐Weyl‐type, described by four orbitals if the SOC is neglected. Thus, the Chern number at R is ±1 × 2 = ±2, which compensate with the Chern number at Γ.

We remark that the definition of **L** is the *atomic* OAM. This differs from the contribution due to self‐rotation of a wave packet in the presence of Berry phase.^[^ ^20^ ^]^ One can show that (Supporting Information) the Berry phase contribution to the orbital moment is given by

(4) mmk=e2ℏIm⟨∂kumk|×Hk−Emk|∂kumk⟩=−12ξmeχvFk^|k|2

where ξ_*m*_ = +1, +2, +1 for *m* = −1, 0, 1, respectively. As shown in ref. [21], this contribution to the orbital moment is different from the Berry curvature. However, as we explicitly show in Equation (2), it is smooth variation of the atomic OAM **L** in **k**‐space by the radial texture that drives the Berry curvature.

While the above discussion on the Berry curvature and the Chern number is for the spinless multifold chiral fermion, when the spin degree of freedom is taken into account, we obtain two spin up and down copies of multifold chiral fermions, which makes the effective Chern number of *C*_*m*_ = 4*m* = −4, 0, + 4. Although the spin also exhibits chirality, it is *derived* from the OAM chirality via SOC. The spin splitting is significantly smaller than the OAM splitting in the band structure, and disappears when the SOC is neglected (Supplementary Information). Therefore, the OAM is the key link between the chirality of crystal structures and the chirality of the OAM texture in **k**‐space.

In particular, the Berry curvature in the topological chiral semimetal CoSi is of *orbital origin*, without requiring SOC for its emergence. Thus, the opposite signs of the OAM chirality can be directly translated to opposite topological charges and the Berry curvature fields in **k**‐space. One of the striking manifestations of the orbital origin of the topological charges in the bulk is the strong OAM polarization of the Fermi arcs, which we confirm in our work. We emphasize that in bulk CoSi, enantiomers A and B have exactly the same band structure. However, the opposite OAM chirality as well as the topological charges suggest that the Fermi arcs at the surface exhibit differently shaped profiles for enantiomers A and B. Thus, measuring Fermi arcs can be a way to distinguish the two enantiomers in an experiment, as discussed in refs. [22, 23, 24, 25]. We also note that the rotation sense of the *helicoid* Fermi arcs, a helix‐like feature in the energy domain, also differs in enantiomers A and B due to the opposite OAM chirality of the multifold chiral fermions in the bulk.

## 3 Detection of the OAM by Momentum Microscopy

To experimentally measure the electronic band structure and the OAM texture, we employed a momentum‐resolved photoemission microscope setup at the NanoESCA beamline of the Elettra synchrotron in Trieste (Italy).^[^ ^26^ ^]^ In a momentum microscope, photoelectrons emitted into the complete solid angle above the sample surface can be collected simultaneously, such that the 2D photoelectron distribution of the in‐plane crystal momentum (*k*_*x*_, *k*_*y*_) is be obtained in a single measurement, covering a wide region over the whole Brillouin zone (BZ).^[^ ^27^, ^28^ ^]^ This is one of the major advantages of the momentum microscope over other techniques such as angle‐resolved photoemission spectroscopy, which necessitate angle‐dependent measurements that alter the experimental geometry, thus complicating the characterization of OAM textures across the entire surface BZ. The photoemission experiments were performed in the geometry schematically shown in Figure 1c. The plane of incidence coincides with the $\bar{\Gamma }-\bar{Y}$ direction of the surface BZ., which thus represents a distinguished plane of high symmetry for the experimental geometry.

To confirm whether the structural chirality reflects in the electronic structure via orbital‐momentum locking, we carried out momentum‐resolved photoemission experiments using left‐ and right‐circularly polarized light (LCP and RCP). The examination of CD in the angular distribution (CDAD) extends beyond the band structure mapping, providing a more comprehensive exploration of the momentum‐resolved Bloch wave function, including orbitals,^[^ ^13^, ^14^, ^15^, ^17^, ^18^ ^]^ Berry curvature,^[^ ^13^, ^16^, ^29^ ^]^ and topological invariants.^[^ ^13^, ^17^, ^30^ ^]^ Moreover, this method allows us to distinguish the handedness of the chiral crystals and their surfaces.^[^ ^31^ ^]^ The OAM contribution in the CDAD^[^ ^32^ ^]^ can be intuitively understood by considering both the helicity of light and the self‐rotation of the Bloch states.^[^ ^15^, ^16^, ^17^ ^]^ In nonmagnetic materials, the combination of time‐reversal and spacial inversion symmetries is a strong condition of orbital quenching, and dictates that OAM is absent for all states.^[^ ^33^ ^]^ The broken symmetry in a chiral crystal, however, can give rise to the presence of OAM at each **k**‐point. Meanwhile, ref. [34] found that the CD asymmetry persists over wide range of photon energies. These previous works suggest that the OAM can be detected in a robust manner by the CD.

We note that circularly polarized light couples not only to an intrinsic chiral system, but also to a chirality introduced by the experimental setup itself.^[^ ^17^, ^35^ ^]^ That is, the incoming photon vector ***q***, the photoelectron momentum ***p***, and the surface normal $\hat{n}$ define a handed coordinate system. When ***q***, $\hat{n}$, and ***p*** are non‐coplanar, a contribution to the circular dichroism thus can arises due to the experimental geometry, even if the sample lacks inherent chirality. The CDAD contribution arising from the experimental geometry vanishes on the *k*_*x*_ = 0 line ($\bar{\Gamma }-\bar{Y}$ direction), where all three vectors, ***q***, ***p***, and $\hat{n}$ lie in the same plane. Therefore, the CDAD along the $\bar{\Gamma }-\bar{Y}$ direction reflects the intrinsic chirality of the electronic states, as no dichroism is induced by experimental geometry.

By using different photon energies, which select different *k*_*z*_ values (see Figure 1d),^[^ ^10^, ^36^, ^37^ ^]^ we predominantly examine either multifold chiral fermion states in the bulk or helicoid Fermi arc states at the surface. At ℏν = 56 eV, we probe a section through the center of the hole pocket at the Γ point of the bulk BZ, where the presence of multifold chiral fermions is anticipated. At ℏν = 78 eV, the *k*_*z*_ cut is positioned between the hole and electron pockets of the Fermi surfaces in the bulk BZ.

## 4 OAM Chirality of Multifold Chiral Fermions

**Figure 2:**
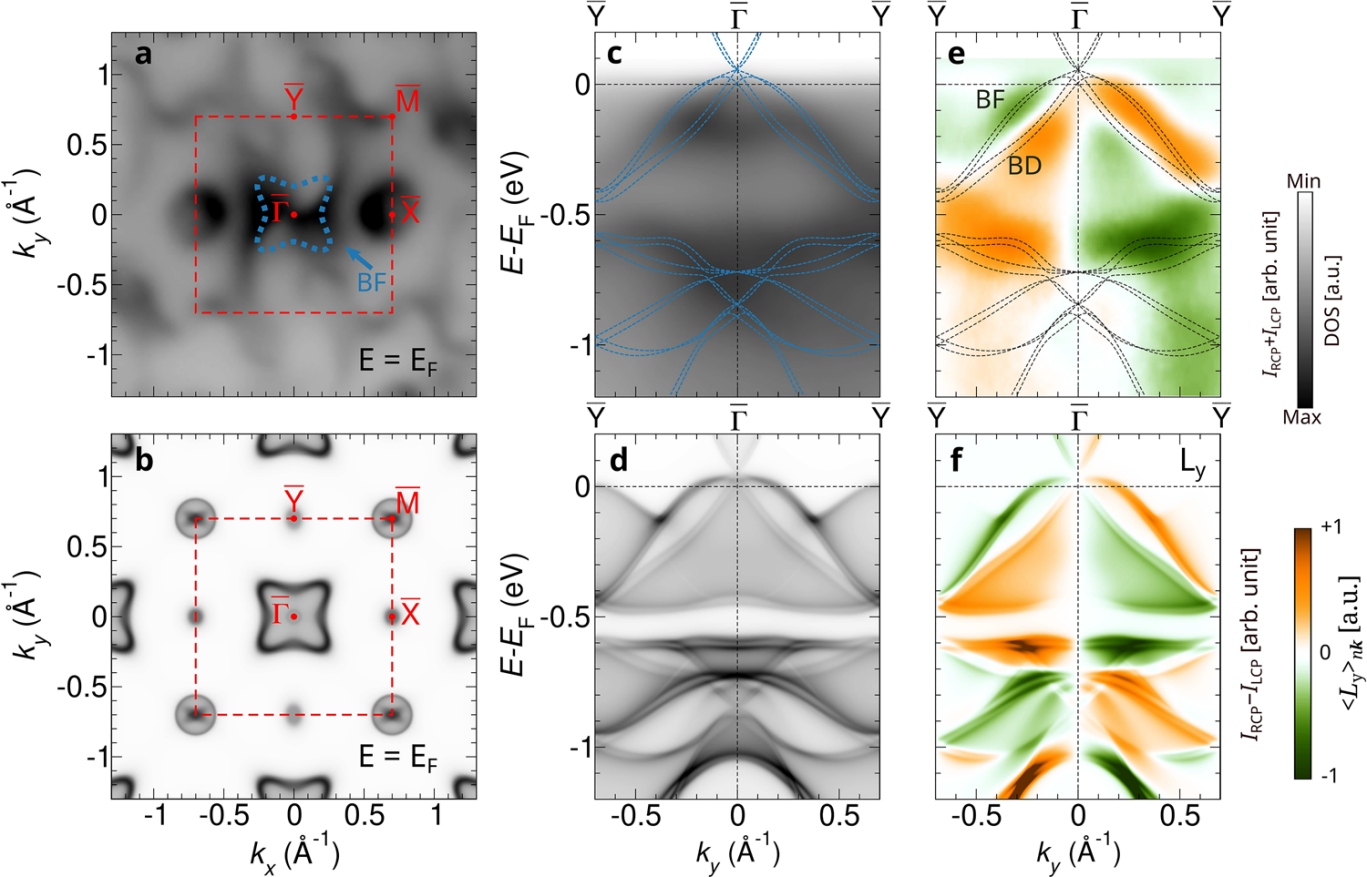
OAM fingerprint of multifold chiral fermions in the bulk. a‐d, Measured photoemission intensity (a,c) and calculated spectral weight (b,d) projected on the surface BZ at the Fermi energy and along the $\bar{Y}-\bar{\Gamma }-\bar{Y}$ path as a function of the energy. e, Measured CD map and f, calculated *L*_*y*_‐projected spectral weight. Bulk flat (BF) and bulk Dirac (BD) bands are indicated in a and e. In c and e, the calculated bulk band structure is shown by dashed lines.

**Figure 3:**
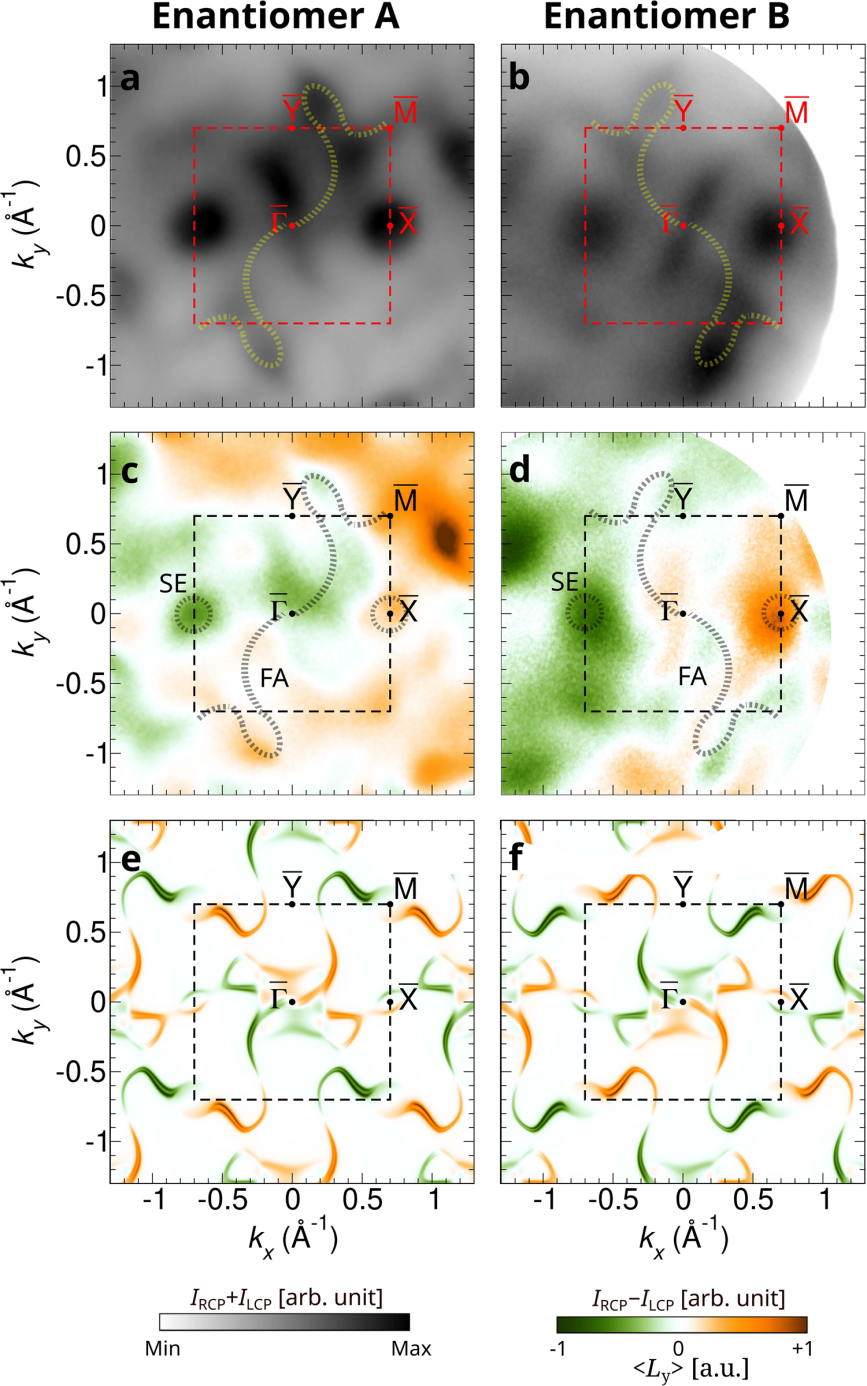
Chirality‐driven orbital texture of helicoid topological Fermi arcs. a,b) The sum of intensities of photoemission momentum maps measured by LCP and RCP light at the Fermi energy for enantiomers A and B. c,d, The measured CD maps for enantiomers A and B. e,f, Calculated spectral weight at a surface with *L*_*y*_ projection, for enantiomers A and B. We denote the Fermi arcs and surface electrons by FA and SE, respectively.

Multifold chiral fermions at Γ are found in the bulk band structure of CoSi (Figure 1b), which can be accessed in our photoemission experiment with a photon energy of 56 eV. Here, we focus on enantiomer A. Figure 2a shows the 2D photoemission intensity map on the surface BZ at the Fermi energy. The intensity is measured by summing up the intensities recorded with LCP and RCP light (*I*_Total_ = *I*_LCP_ + *I*_RCP_). As marked by the blue dashed line, bulk flat (BF) bands of the chiral fermions are observed at E_F_. This feature is also found in the calculated spectral weight shown in Figure 2b. While bulk states exhibit nearly four‐fold rotation‐symmetric Fermi surface, the B20 crystal has only two‐fold screw rotation along (001),^[^ ^38^, ^39^, ^40^ ^]^ strictly speaking. This explains slightly different weights at $\bar{X}$ and $\bar{Y}$ in the theoretical calculation (Figure 2b). This asymmetry manifests more drastically in the experimental data (Figure 2a), which we attribute to surface states. Moreover, we also find the topological helicoid arcs connecting $\bar{\Gamma }$ and $\bar{M}$, which are two‐fold rotation‐symmetric. Here, we focus mainly on the multifold chiral fermions in the bulk, while features of the surface state are revisited in Figure 3. In Figure 2c. Photoemission intensities along the $\bar{Y}-\bar{\Gamma }-\bar{Y}$ direction are shown as a function of the energy, together with the calculated bulk band structure (dashed lines). In comparison with the computed spectral weight projected on the surface BZ (Figure 2d), a good agreement between theory and experiment is obtained.

The OAM of the multifold chiral fermions can be assessed by the CD (*I*_CD_ = *I*_LCP_ − *I*_RCP_). As shown in Figure 2e, the pronounced CD signal along the $\bar{Y}-\bar{\Gamma }-\bar{Y}$ direction signifies the OAM, suggesting substantial contributions to the Berry curvature in momentum space. Notably, substantial CD signals within the optical plane reveal a significant OAM *L*_*y*_, parallel to the momentum *k*_*y*_. The sign of the CD signal is an odd function of *k*_*y*_, being consistent with the monopole‐like radial texture of the OAM, giving rise to the chirality (**k** · **L**) of the multifold chiral fermions. We find that the BF and bulk Dirac (BD) bands exhibit the opposite OAM chirality. We corroborate these findings by theory as well. Figure 2f shows the calculated *L*_*y*_‐projected spectral weight on the surface BZ, which is in excellent agreement with the CD measured in our experiment (Figure 2e). The weights for the other components of the OAM are shown in the Supplementary Information. We emphasize that the OAM polarization of the helicoid Fermi arc is of nonrelativistic origin, which depends on the crystal chirality. The spin texture can arise with the aid of SOC, but since SOC is much weaker compared to the crystal‐field potential in this material, the spin splittings are much smaller, i.e. spin up and down bands are nearly degenerate (Supplementary Information).

## 5 OAM Texture of Helicoid Topological Fermi arcs

At a photon energy of 78 eV, the photoemission signal from the bulk electron and hole pockets can be reduced (Figure 1d), which allows us to better analyze the surface states. The total intensity map (*I*_Tot_ = *I*_LCP_ + *I*_RCP_) over the surface BZ at the Fermi energy is shown in Figures 3a and 3b, for enantiomers A and B, respectively. We clearly observe the absence of the four‐fold rotation symmetry, as the surface does not preserve cubic symmetry anymore. Importantly, the “direction” of symmetry breaking is opposite between enantiomers A and B. Thus, although the bulk spectra are identical for both enantiomers, examining the surface allows to distinguish them.

One of the most distinctive spectral features at the surface of CoSi is the emergence of helicoid topological Fermi arcs. They originate from the difference of the Chern numbers of the multifold chiral fermions at the Γ and R points in the bulk (Figure 1b). Thus, the presence of the Fermi arcs is guaranteed by the bulk‐boundary correspondence principle in the surface BZ between $\bar{\Gamma }$ and $\bar{M}$. These points are maximally separated in momentum space (Table 1). In general, how the chiral charges are connected by Fermi arcs depends on details of the microscopic interactions at the surface. Interestingly, this connection is different for enantiomers A and B. As indicated by dashed lines in Figure 3a and 3b, the Fermi arcs of enantiomers A and B connect between Γ and (+ 0.7, +0.7) Å^−1^ and between Γ and (− 0.7, +0.7) Å^−1^ which reflects the breaking of the screw rotation symmetry at the surface. Despite this difference, the Fermi arcs of enantiomers A and B are related by mirror reflection along the [100] axis.

Another striking difference between the Fermi arcs of enantiomer A and B is their OAM polarization. In Figure 3c and 3d, the CD (*I*_CD_ = *I*_LCP_ − *I*_RCP_) is shown over the surface BZ, measured on the surfaces of enantiomers A and B, respectively. The results show that the Fermi arcs exhibit a pronounced OAM polarization, which shows a clear difference between enantiomers A and B, related by a mirror reflection along the *a*‐axis.

Previous studies demonstrate that the observed CD in photoemission experiments from the surface states of both topological materials and elementary metals is attributed to the existence of local OAM.^[^ ^14^, ^15^ ^]^ Their findings reveal that CD is proportional to the inner product of the light propagation and OAM vectors for in‐plane component (*L*_*y*_ in our case).

To understand the difference of the Fermi arcs in enantiomers A and B, in Figures 3e and 3f, we show the calculated results of the *L*_*y*_‐projected spectral weights, respectively. The surface spectrum and the OAM polarization are calculated by the surface Green's function technique for a semi‐infinite geometry, based on the Wannier representation of the bulk periodic electronic structure. In general, we find that the overall shapes of the Fermi arcs agree with the experimental results (Figures 3a and 3b); the calculation clearly shows that the Fermi arcs connect between $\bar{\Gamma }$ and $\bar{M}$, and exhibit an opposite rotation sense in enantiomers A and B.

In general, the OAM textures of helicoid Fermi arcs exhibit richer features compared to those of bulk states. Detailed results of the first‐principles calculation of the surface OAM texture can be found in Supplementary Information. The surface OAM texture has both *intrinsic* and *extrinsic* origins. The intrinsic origin is due to the distinct topological charges carried by multifold chiral fermions in the bulk, depending on the crystal chirality. This comprises ‘radial’ OAM texture (**L**∥**k**) of the helicoid Fermi arcs. Despite the similarity to the bulk OAM texture, however, we find that the surface OAM polarization is substantially larger than that in the bulk. The extrinsic origin is due to modified potential at surfaces. This includes a Rashba‐type OAM texture ($L∥\hat{z}\times k$), induced by the potential gradient along *z*. We also find a *L*_*z*_ texture, which originates from the potential gradient in the *xy* plane.

The calculated *L*_*y*_ agrees well with the experimental result (Figures 3c and 3d) for the Fermi arcs. In particular, for the states near $\bar{M}$, the sign change of *L*_*y*_ is well reproduced at the Brillouin zone boundary. However, a quantitative comparison of *L*_*y*_ is challanging for the states near $\bar{\Gamma }$. This can be understood by the large spectral weight of the bulk states: as schematically illustrated in Figure 1d, the hole pocket near $\bar{\Gamma }$ is substantially larger than the electron pocket near $\bar{M}$, which is also confirmed by our first‐principles calculation (Supplementary Information). Furthermore, we remark that in general the OAM polarization depends strongly on the electric polarization at a surface.^[^ ^41^, ^42^, ^43^ ^]^ Quantitatively comparing the theoretical and experimental values of the OAM thus is a challenging task, since the detailed sign and magnitude of the orbital polarization may significantly change depending on the condition at the surface, whose assumption in the theory generally differs from the condition in the experiment. For example, the atoms at a surface may undergo a structural relaxation, which is not considered in the calculations.

Nonetheless, the *symmetry relation* between the OAM textures of enantiomers A and B, in terms of the mirror reflection along the *a*‐axis, is robust in both theory and experiment. That is, *L*_*y*_(*k*_*x*_, *k*_*y*_) in enantiomer A is equal to −*L*_*y*_(− *k*_*x*_, *k*_*y*_). Meanwhile, we also remark that the SOC in this system is small, resulting in a spin splitting of the Fermi arcs that is far too small to be detected in the experiments (Supplementary Information). The OAM textures of the Fermi arcs are a direct consequence of the crystal‐field potential, not the SOC.

Beside the helicoid Fermi arcs, also other surface states are observed: a disk shaped state at the $\bar{X}$ point forms a surface electron (SE) pocket as indicated in Figure 3c,d. These states are a usual closed loop and therefore of a non‐chiral origin, which does not carry a topological charge, e.g., the orbital Rashba interaction at the surface. Thus, *L*_*y*_ polarizations at $\bar{X}$ are the same for both enantiomers A and B, which is consistent with theoretical calculation (Supporting Information). Interestingly, the SE pocket is not pronounced in the spectrum obtained by a semi‐infinite slab calculation, but a fully consistent first‐principles calculation of a finite slab reproduces the large spectral weight at $\bar{X}$ (Supplementary Information). This suggests the SE states rely sensitively on the boundary condition at the surface, and thus are independent from the band topology in the bulk.

## 6 Concluding Remarks and Outlook

In this work, we have shown the crucial role of the OAM as the key link intertwining crystal chirality, electronic chirality and band topology in the topological chiral crystal CoSi by momentum microscopy experiments and first‐principles calculations. In the bulk, the radial OAM texture is not only a characteristic feature of multifold chiral fermions but also the origin of a high Chern number. As the orbital degree of freedom directly interacts with the crystal‐field potential, enantiomers A and B exhibit an opposite sign of the OAM texture, Berry curvature, and the Chern number of their electronic bands. The nontrivial topology of the multifold chiral fermions in the bulk manifests at the surface, where helicoid Fermi arcs connect the surface projections of the two bulk topological charges. We demonstrate the orbital origin of the helicoid Fermi arcs by observing a pronounced OAM texture, which depends on the crystal chirality.

Our work bridges the two separate but important areas of chiral chemistry/physics and topological materials. On the one hand, the notion of atomic and molecular orbitals is the natural language in chiral chemistry, as they couple directly to chiral structures. However, the orbitals have been regarded as mainly responsible for the stability of molecules and materials and not considered as the “information” or the degree of freedom. On the other hand, in the study of topological materials, the role of the orbital degree of freedom has been overlooked; historically most early findings have been associated with SOC and the spin‐momentum locking. While this early development was driven by analogy with particle physics, as we pointed out, the multifold chiral fermion, which originates from the orbital degree of freedom, finds no analogy among elementary particles. Therefore, in the new interdisciplinary field of chiral physics in topological materials, it is encouraged to investigate the manifestations of the OAM further, e.g. in transport and optical phenomena, as well as reaction dynamics, in a unified framework. Promoting the OAM as a foundation for topological characterization is another pending challenge, which has to be addressed in the future. Chirality emerges as an analog of spin or magnetization for spin and charge derived topologies – a new Ising‐like variable for topological classification rooting in OAM of electronic states. In this context the OAM‐powered chirality‐sensitive Fermi arcs will be crucial for understanding linear and non‐linear (magneto‐)trasport orbitally‐flavored phenomena arising at the boundaries between domains with different crystal chirality.

In this context, our findings may resonate with recent developments of “orbitronics”, which aims to exploit the OAM or the orbital degree of freedom of electrons as an information carrier in next‐generation electronic devices.^[^ ^33^ ^]^ Orbital‐momentum locking present in both bulk and surface states makes topological chiral crystals an intriguing material platform to discover novel quantum transport phenomena beyond the orbital Hall effect and the orbital Edelstein effect.^[^ ^44^, ^45^ ^]^ For example, the orbital texture of the helicoid Fermi arcs may be engineered for orbitronic devices and harnessed by converting between OAM and the electron spin for spintronic devices. Note that the idea of utilizing the Fermi arc of a Weyl semimetal for generating non‐equilibrium spin was proposed by Johansson *et al.*,^[^ ^46^ ^]^ which may be generalized to the case of the OAM. The interplay of structural chirality with electronic OAM at the surface of chiral metals presents other exciting possibilities for orbitronics realizations, such as, e.g., excitation of chiral phonons^[^ ^47^, ^48^ ^]^ by electrical currents and orbital relaxation, OAM‐mediated structural chirality switching by circularly polarized light or effects of orbital filtering in magneto‐resistance type of transport applications.

## 7 Experimental Section

### Sample Preparation

High‐quality single crystals were grown by the modified Bridgeman method with the help of an optical floating‐zone furnace as described in ref. [49, 50] and polished on the (001) surface. Since the Co and Si atoms are strongly bonded by multiple covalent bonds in three dimensions and the high symmetry surfaces are not cleavage planes, we performed Ar^+^ ion sputtering and annealing in the preparation chamber of the NanoESCA beamline in order to get a clean sample surface instated of cleaving the sample. Referring to the cleaning procedure of the transition‐metal monosilicides FeSi^[^ ^51^, ^52^ ^]^ and MnSi,^[^ ^53^ ^]^ we obtained the best condition for the in situ surface preparation of CoSi: Ar^+^ ion sputtering with an energy of 2 keV and annealing at *T* = 680°C.

### Momentum Microscopy Measurements

Momentum‐resolved photoelectron spectroscopy experiments were carried out at the NanoESCA beamline^[^ ^26^ ^]^ of the Elettra synchrotron in Trieste (Italy), using left‐ and right‐circularly polarized light, without changing the overall experimental geometry. All measurements were performed while keeping the sample at a temperature of 130 K. Photoelectrons emitted into the complete solid angle above the sample surface were collected using a momentum microscope.^[^ ^27^, ^54^ ^]^ The momentum microscope directly forms an image of the distribution of photoelectrons as function of the lateral crystal momentum (*k*_*x*_, *k*_*y*_) that is recorded on an imaging detector.^[^ ^27^, ^54^ ^]^

### First‐Principles Calculations

The electronic band structure and the OAM texture are computed by the Wannier interpolation method from first‐principles, which consists of three steps of calculation. First, we converge the charge density and potential by self‐consistent density functional theory calculation. Here, we employ the FLEUR code,^[^ ^55^ ^]^ in which the full‐potential linearly augmented plane wave method^[^ ^56^ ^]^ is implemented. The exchange and correlation effects are treated within the generalized gradient approximation by using the Perdew–Burke–Ernzerhof functional.^[^ ^57^ ^]^ The lattice constant of the simple cubic structure is set 8.38*a*_0_, where *a*_0_ is the Bohr radius, wherein the atom positions are listed in Table 2. We set the maximum of the harmonic expansion in the muffin‐tin sphere *l*_max_ = 12 and the muffin‐tin radii of both Co and Si atoms *R*_MT_ = 2.13*a*_0_. The plane wave cutoff is set $K_{\max}=5.0a_{0}^{-1}$. We sample **k**‐points from the Monkhorst‐Pack mesh of 16 × 16 × 16. The SOC is considered in the fully relativistic manner by the second variation scheme.

**Table 2:** The positions of Co and Si atoms in the fractional coordinate of the simple cubic unit cell.

Atom	Fractional coordinate
Co‐1	(0.1451, 0.1451, 0.1451)
Co‐2	(0.3549, 0.8549, 0.6451)
Co‐3	(0.6451, 0.3549, 0.8549)
Co‐4	(0.8549, 0.6451, 0.3549)
Si‐1	(0.1568, 0.3432, 0.6568)
Si‐2	(0.3432, 0.6568, 0.1568)
Si‐3	(0.6568, 0.1568, 0.3432)
Si‐4	(0.8432, 0.8432, 0.8432)

Second, we construct the maximally localized Wannier functions from the converged Kohn–Sham states from the first step. As the initial guess, we project the Kohn–Sham states onto *d*_*xy*_, *d*_*yz*_, *d*_*zx*_, $d_{z^{2}}$, $d_{x^{2}-y^{2}}$ orbitals on Co sites and *s*, *p*_*x*_, *p*_*y*_, *p*_*z*_ orbitals on Si sites. The spread of Wannier functions are iteratively minimized by using the WANNIER90 code.^[^ ^58^ ^]^ For the disentanglement step, we set the frozen energy window such that its maximum is 2 eV above the Fermi energy. The Hamiltonian, position, spin, and OAM operators, which are initially evaluated in the Kohn‐Sham basis, are transformed into the Wannier basis by using the interface between the FLEUR and WANNIER90 codes.^[^ ^59^ ^]^ The OAM is evaluated within the atom‐centered approximation.^[^ ^60^ ^]^

Third, we use the home‐built code ORBITRANS to evaluate the OAM expectation value of the bulk band structure as well as the OAM‐projected spectral weight of the in the semi‐infinite geometry. For the latter, we implement the surface Green's function method for the consideration of the semi‐infinite slab by following the scheme in ref. [61]. For the Wannier interpolation along high‐symmetry points, we use 300 points, and for the plots on the surface BZ, we use a 200 × 200 meshgrid.

## Conflict of Interest

The authors declare no conflict of interest.

## Author Contributions

KH, YJC, XLT, KHOY, YHS, JC, YHS, IC, VF, CT performed the photoemission experiments. KH, XLT, KHOY, YHS, CJ, YHC, YJC performed the initial surface preparation and characterization under the supervision of MTL, CT. KH analyzed the experimental data with suggestions by YJC, XLT and supervised by CT. DG developed the first‐principles method to compute the OAM texture in the bulk and surface and performed calculations under the supervision of YM. SG performed band structure calculation under the supervision of SB. GJS, XLH, KHOY synthesized and characterized the samples. KH, YJC, DG drafted the manuscript under supervision from CT. CT designed and coordinated the research together with MTL, CMS. All authors discussed the results and contributed to improving the manuscript.

## Supporting information


Supplemental Movie S1

## Data Availability

The data that support the findings of this study are available from the corresponding author upon reasonable request.
