# Supplementary material for: Orbital Topology of Chiral Crystals for Orbitronics
Source: Adv Mater. 2025 May 2;37(27):2418040. doi: 10.1002/adma.202418040 (PMC12243713; doi:10.1002/adma.202418040)
Supplement: Supplementary file 1 — Supplemental Movie S1 [file ADMA-37-2418040-s001.pdf]

ADVANCED MATERIALS

Supporting Information

for *Adv. Mater.*, DOI 10.1002/adma.202418040

Orbital Topology of Chiral Crystals for Orbitronics

*Kenta Hagiwara*, Ying-Jiun Chen*, Dongwook Go*, Xin Liang Tan, Sergii Grytsiuk, Kui-Hon Ou Yang, Guo-Jiun Shu, Jing Chien, Yi-Hsin Shen, Xiang-Lin Huang, Iulia Cojocariu, Vitaliy Feyer, Minn-Tsong Lin, Stefan Blügel, Claus Michael Schneider, Yuriy Mokrousov and Christian Tusche**

# Supplementary Information for
# "Orbital Topology of Chiral Crystals for Orbitronics"

Kenta Hagiwara, Ying-Jiun Chen, Dongwook Go, Xin Liang Tan, Sergii Grytsiuk, Kui-Hon Ou Yang, Guo-Jiun Shu, Jing Chien, Yi-Hsin Shen, Xiang-Lin Huang, Iulia Cojocariu, Vitaliy Feyer, Minn-Tsong Lin, Stefan Blügel, Claus Michael Schneider, Yuriy Mokrousov, Christian Tusche

## 1. Summary of the features of the band structure and spin and orbital textures

- The orbital texture is radial near $\Gamma$ and $\mathrm{R}$, where multifold chiral fermions reside. Along $k_x$ direction, only $L_x$ is finite. Similarly, Along $k_y(k_z)$ direction, only $L_y(L_z)$ is finite. See Figs. S1 and S2.
- The radial textures of enantiomers A and B exhibit the opposite chirality, respectively at $\Gamma$ and $\mathrm{R}$. That is, the topological charges of the multifold chiral fermions are the opposite in enantiomers A and B. Compare Fig. S1 and Fig. S2.
- For each enantiomer A or B, the chiralities of the radial orbital textures are opposite at $\Gamma$ and $\mathrm{R}$. This implies that the multifold chiral fermions at $\Gamma$ and $\mathrm{R}$ carry the opposite topological charges. Compare Figs. S1**a-c** with Fig. S1**d-f** and Figs. S2**a-c** with Fig. S2**d-f**.
- On the surface Brillouin zone, the $L_x$ and $L_y$ clearly show the radial textures, with the opposite chiralities at $\bar{\Gamma}$ and $\bar{\mathrm{M}}$, see Fig. S3**a-c** and Fig. S4**a-c** for the projected bulk textures for enantiomers A and B, respectively. The orbital textures are opposite in enantiomers A and B. Since $L_z$ is an odd function of $k_z$, the $L_z$ texture is vanishes in the bulk, when projected onto the surface Brillouin zone.
- At surfaces, the topological Fermi arcs appear. Their connectivities are different at enantiomers A and B. For enantiomer A, the Fermi arcs connect between $\bar{\Gamma}$ and $(+\pi,+\pi)$ and between $\bar{\Gamma}$ and $(-\pi,-\pi)$ (Figs. S3**d-f**). On the other hand, for enantiomer B, the Fermi arcs connect between $\bar{\Gamma}$ and $(+\pi,-\pi)$ and between $\bar{\Gamma}$ and $(-\pi,+\pi)$ (Figs. S4**d-f**).
- The topological Fermi arcs exhibit strong orbital polarization. The orbital plarizations in enantiomers A and B are also different. Compare Figs. S3**d-f** with **Figs. S4d-f**.
- A finite-slab calculation is performed to check the robustness of the semi-infinite slab calculation. They generally agree well, especially the connectivity of the topological Fermi arcs and their orbital textures. Compare Figs. S4**d-f** with Figs. S5**d-f**. This implies the topological origin of the Fermi arcs, which is insensitive to the detailed boundary condition at a surface.
- On the other hand, the finite-slab calculation shows significant weight at $\bar{\mathrm{X}}$, see Figs. S5**d-f**. The difference from the semi-infinite slab calculation suggests that this is a surface state of non-topological origin.
- The band structure of CoSi also exhibits radial spin polarization, see Fig. S6 for the results of enantiomer A. However, the spin-dependent energy splittings are an order of magnitude smaller than those for the orbital, which is far too small to be resolved in our experiment.

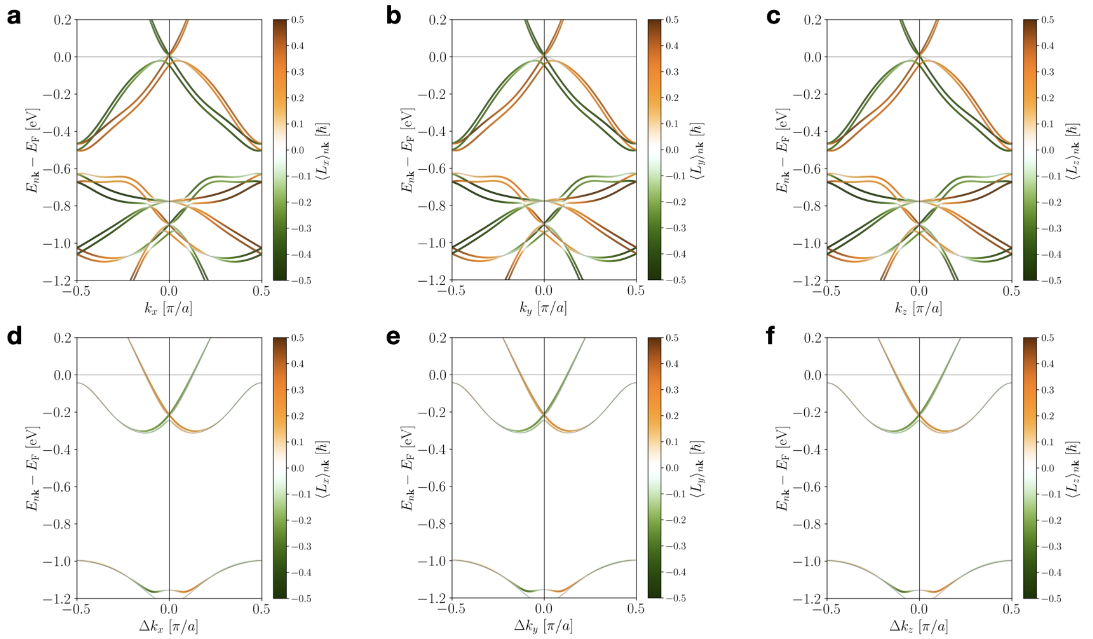

FIG. S1. **Band structure and orbital texture of enantiomer A in the bulk. a-c,** The results for $L_x$, $L_y$, $L_z$ textures along $k_x$, $k_y$, $k_z$ axes, near $\Gamma$. **d-f,** The analogous results near $\mathrm{R} = (\pi, \pi, \pi)/a$.

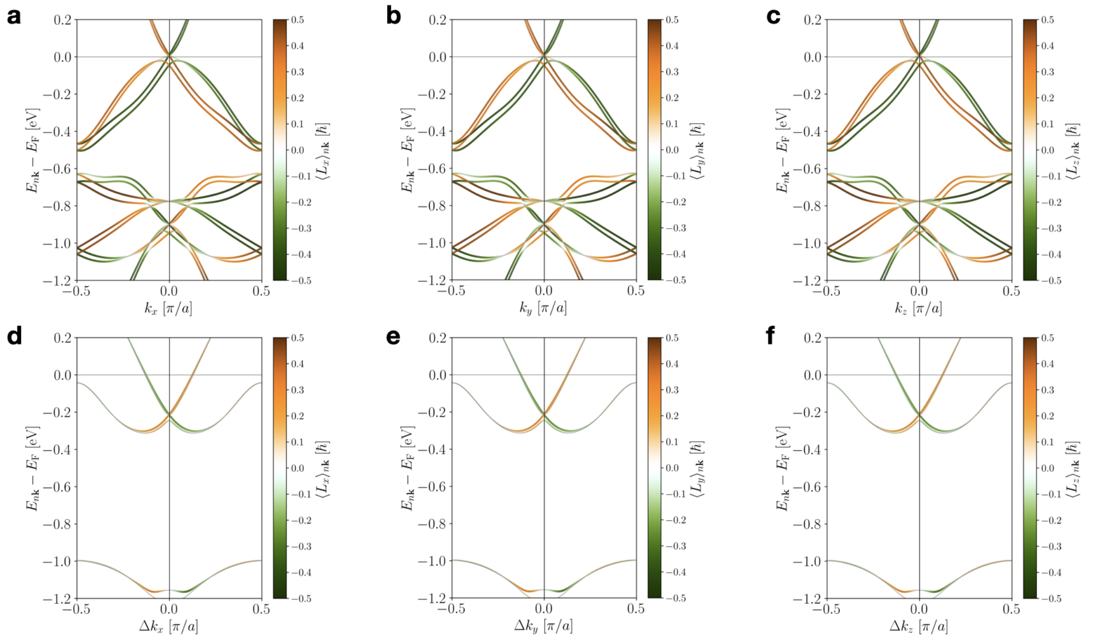

FIG. S2. **Band structure and orbital texture of enantiomer B in the bulk. a-c,** The results for $L_x$, $L_y$, $L_z$ textures along $k_x$, $k_y$, $k_z$ axes, near $\Gamma$. **d-f,** The analogous results near $\mathrm{R} = (\pi, \pi, \pi)/a$.

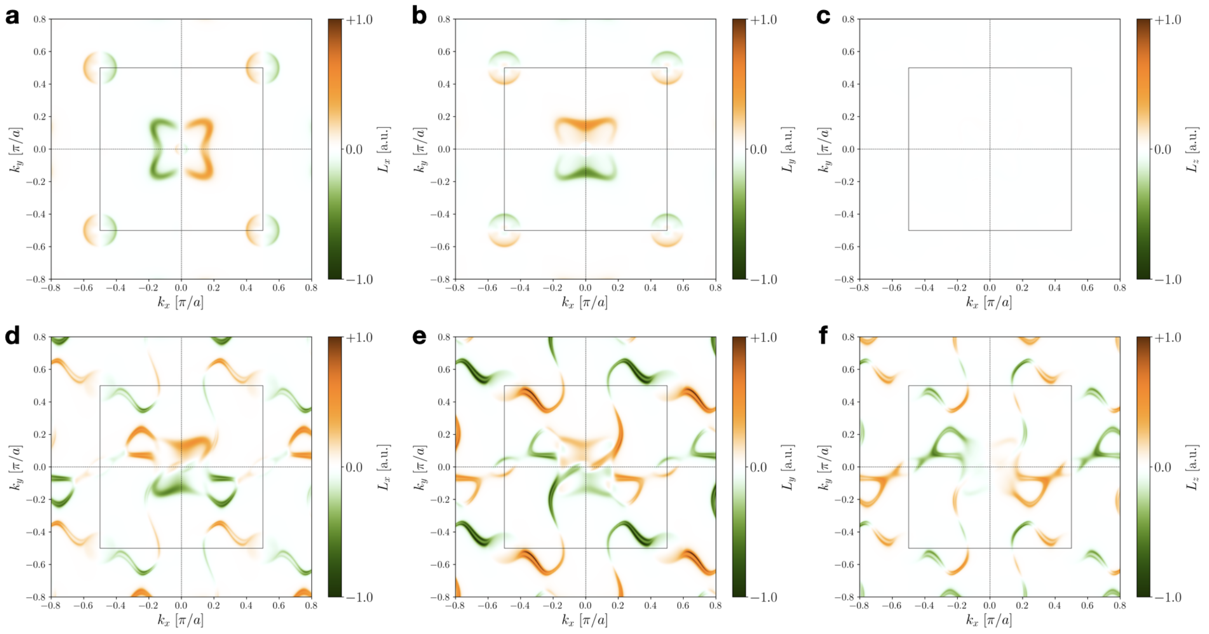

FIG. S3. **Orbital texture of enantiomer A on the surface Brillouin zone calculated for a semi-infinite slab. a-c,** $L_x$, $L_y$, $L_z$ textures in the bulk. **d-f,** $L_x$, $L_y$, $L_z$ textures on a surface (top 24 atoms from the vacuum).

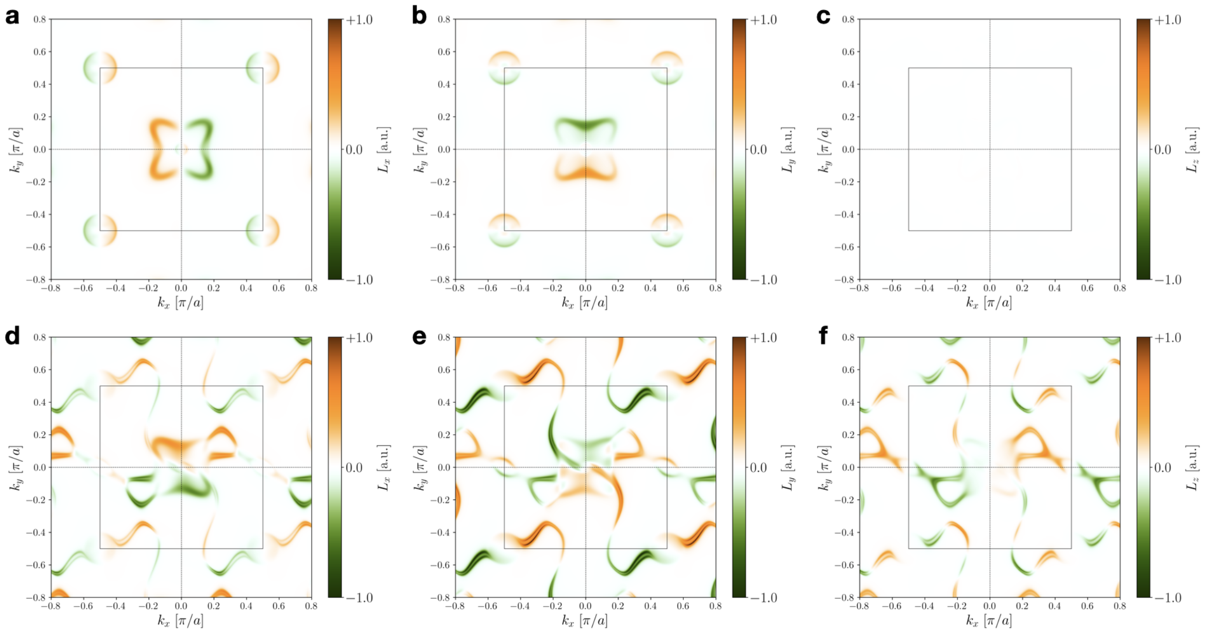

FIG. S4. **Orbital texture of enantiomer B on the surface Brillouin zone calculated for a semi-infinite slab. a-c,** $L_x$, $L_y$, $L_z$ textures in the bulk. **d-f,** $L_x$, $L_y$, $L_z$ textures on a surface (top 24 atoms from the vacuum).

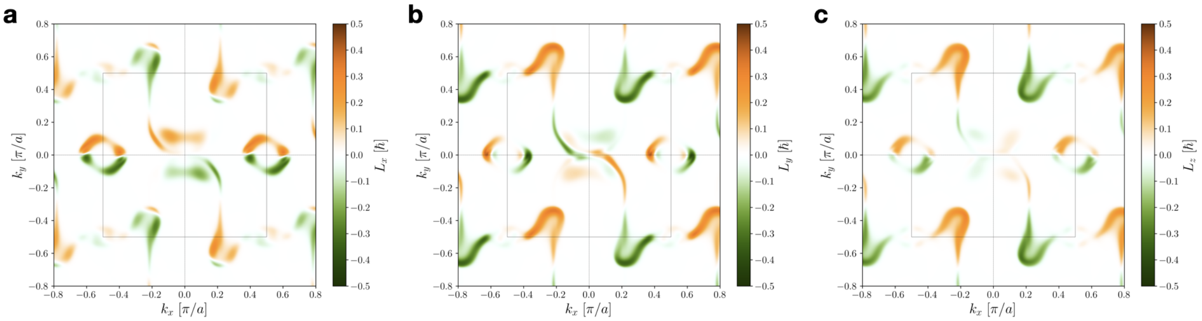


FIG. S5. **Orbital texture of enantiomer B on the surface Brillouin zone calculated for a infinite slab. a-c,** $L_x$, $L_y$, $L_z$ textures projected on the top 20 atoms out of the 40 atomic layers of the slab.

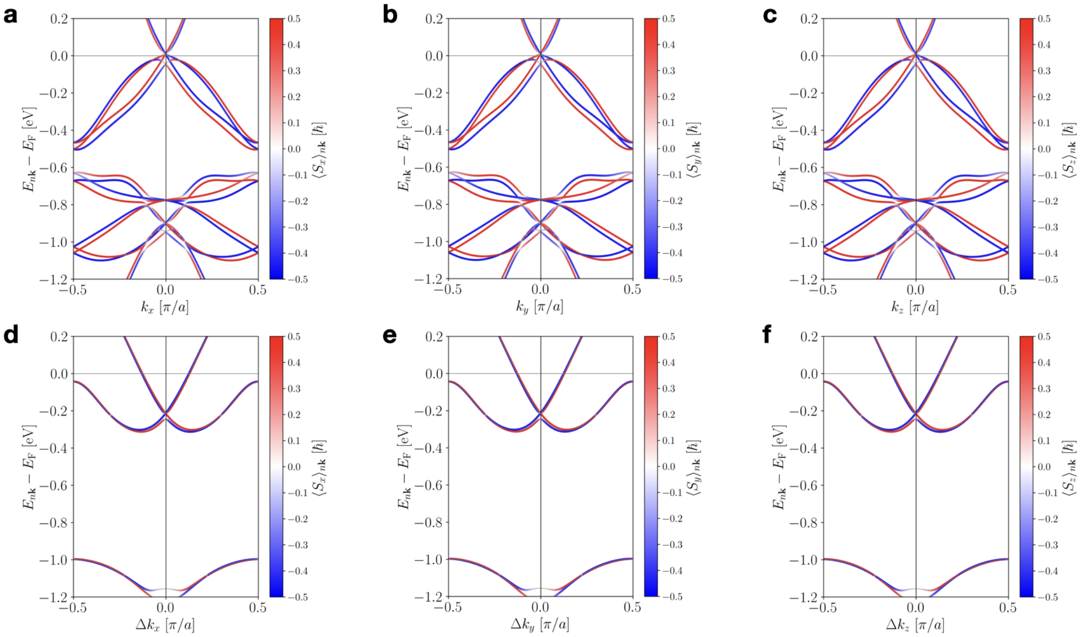


FIG. S6. **Band structure and spin texture of enantiomer A in the bulk. a-c,** The results for $S_x$, $S_y$, $S_z$ textures along $k_x$, $k_y$, $k_z$ axes, near $\Gamma$. **d-f,** The analogous results near $\mathrm{R} = (\pi, \pi, \pi)/a$.

## 2. Relation between the orbital angular momentum and the Berry curvature of the multifold chiral fermion

### 2.1. Model Hamiltonian

The low-energy Hamiltonian for the $l_{\text{eff}} = 1$ chiral fermion is

$$\mathcal{H}_{\mathbf{k}} = \chi v_{\text{F}} \mathbf{L} \cdot \mathbf{k}, \tag{S1}$$

in the lowest order in $\mathbf{k}$, the crystal momentum measured from the crossing. Here, $\chi = \pm 1$ is the chirality, determined by the crystal chirality. The orbital angular momentum (OAM) operator $\mathbf{L}$ for a $l_{\text{eff}} = 1$ is isomorphic to and represented by the OAM of a $p$ electron. The matrix representations of $L_x$, $L_y$, and $L_z$ in the $p_x$, $p_y$, $p_z$ orbital basis are written as

$$L_x = \hbar \begin{pmatrix} 0 & 0 & 0 \\ 0 & 0 & -i \\ 0 & +i & 0 \end{pmatrix}, \; L_y = \hbar \begin{pmatrix} 0 & 0 & +i \\ 0 & 0 & 0 \\ -i & 0 & 0 \end{pmatrix}, \; L_z = \hbar \begin{pmatrix} 0 & -i & 0 \\ +i & 0 & 0 \\ 0 & 0 & 0 \end{pmatrix}, \tag{S2}$$

Meanwhile, $v_{\text{F}}$ is the Fermi velocity, which is assumed to be a constant.

Because the Hamiltonian [Eq. (S1)] is rotationally invariant, we adopt the angular representation in terms of a radial and two tangential states,

$$|u_{p_r\mathbf{k}}\rangle = \sin\theta\cos\phi\,|u_{p_x\mathbf{k}}\rangle + \sin\theta\sin\phi\,|u_{p_y\mathbf{k}}\rangle + \cos\theta\,|u_{p_z\mathbf{k}}\rangle\,, \tag{S3a}$$
$$|u_{p_\theta\mathbf{k}}\rangle = \cos\theta\cos\phi\,|u_{p_x\mathbf{k}}\rangle + \cos\theta\sin\phi\,|u_{p_y\mathbf{k}}\rangle - \sin\theta\,|u_{p_z\mathbf{k}}\rangle\,, \tag{S3b}$$
$$|u_{p_\phi\mathbf{k}}\rangle = -\sin\phi\,|u_{p_x\mathbf{k}}\rangle + \cos\phi\,|u_{p_y\mathbf{k}}\rangle\,, \tag{S3c}$$

where $\theta$ and $\phi$ are the polar and azimuthal angles of $\mathbf{k}$, defined by

$$\theta = \cos^{-1}\left(\frac{k_z}{\sqrt{k_x^2 + k_y^2 + k_z^2}}\right), \tag{S4a}$$
$$\phi = \tan^{-1}\left(\frac{k_y}{k_x}\right), \tag{S4b}$$

and $u_{p_\alpha\mathbf{k}}$ ($\alpha = x, y, z$) is the periodic part of the $p_\alpha$ character Bloch state. With these, the eigenstates of the Hamiltonian are concisely written as

$$|u_{p_0\mathbf{k}}\rangle = |u_{p_r\mathbf{k}}\rangle\,, \tag{S5a}$$
$$|u_{p_{+1}\mathbf{k}}\rangle = \frac{1}{\sqrt{2}}\left(|u_{p_\theta\mathbf{k}}\rangle + i\,|u_{p_\phi\mathbf{k}}\rangle\right), \tag{S5b}$$
$$|u_{p_{-1}\mathbf{k}}\rangle = \frac{1}{\sqrt{2}}\left(|u_{p_\theta\mathbf{k}}\rangle - i\,|u_{p_\phi\mathbf{k}}\rangle\right), \tag{S5c}$$

where the band index $p_m$ is labeled by the orbital quantum number for the quantization axis along $\mathbf{k}$,

$$\mathbf{L}\cdot\hat{\mathbf{k}}\,|u_{p_m\mathbf{k}}\rangle = \hbar m\,|u_{p_m\mathbf{k}}\rangle\,, \tag{S6}$$

because

$$[\mathbf{L}\cdot\hat{\mathbf{k}},\ \mathcal{H}_{\mathbf{k}}] = 0. \tag{S7}$$

The energy eigenvalues are given by

$$\mathcal{E}_{p_m\mathbf{k}} = \chi v_{\text{F}} \hbar m \sqrt{k_x^2 + k_y^2 + k_z^2}. \tag{S8}$$

### 2.2. Berry Curvature and Chern number

The definition of the Berry curvature for $p_m$ band is

$$\mathbf{\Omega}^{p_m}(\mathbf{k}) = -\text{Im}\left[\langle\partial_{\mathbf{k}} u_{p_m\mathbf{k}}| \times |\partial_{\mathbf{k}} u_{p_m\mathbf{k}}\rangle\right]. \tag{S9}$$

Utilizing the rotational invariance of the Hamiltonian [Eq. (S1)], the Berry curvature field in an arbitrary direction can be constructed by calculating only one component. Thus, let us focus only on the $z$ component

$$\Omega_z^{p_m}(\mathbf{k}) = -2\mathrm{Im}\left[\langle \partial_{k_x} u_{p_m\mathbf{k}} | \partial_{k_y} u_{p_m\mathbf{k}}\rangle\right]. \tag{S10}$$

For $p_0$ band, it is easy to show that the Berry curvature is zero by noticing that all the coefficients in the radial state [Eq. (S3a)] are *real*. Their $\mathbf{k}$-derivatives produce only real numbers, and thus, the Berry curvature, given as $\mathrm{Im}[\cdots]$ must be zero.

Now let us calculate the Berry curvature for $p_{\pm1}$ band,

$$\Omega_z^{p_{\pm1}}(\mathbf{k}) = -2\mathrm{Im}\left[\langle \partial_{k_x} u_{p_{\pm1}\mathbf{k}} | \partial_{k_y} u_{p_{\pm1}\mathbf{k}}\rangle\right] \tag{S11a}$$

$$= -\mathrm{Im}\left[\langle \partial_{k_x} u_{p_\theta\mathbf{k}} | \partial_{k_y} u_{p_\theta\mathbf{k}}\rangle + \langle \partial_{k_x} u_{p_\phi\mathbf{k}} | \partial_{k_y} u_{p_\phi\mathbf{k}}\rangle\right]$$
$$\mp\mathrm{Re}\left[\langle \partial_{k_x} u_{p_\theta\mathbf{k}} | \partial_{k_y} u_{p_\phi\mathbf{k}}\rangle - \langle \partial_{k_x} u_{p_\phi\mathbf{k}} | \partial_{k_y} u_{p_\theta\mathbf{k}}\rangle\right]. \tag{S11b}$$

Because all the coefficients in the tangential states [Eqs. (S3b) and (S3c)] are real, $\mathrm{Im}[\cdots]$ on the first line in Eq. (S11b), is zero, and only $\mathrm{Re}[\cdots]$ in the second line makes finite contribution. For this, let us evaluate the $\mathbf{k}$-derivative of the two tangential states [Eqs. (S3b) and (S3c)],

$$|\partial_{\mathbf{k}} u_{p_\theta\mathbf{k}}\rangle = -(\partial_{\mathbf{k}}\theta)\left[\sin\theta\cos\phi\,|u_{p_x\mathbf{k}}\rangle + \sin\theta\sin\phi\,|u_{p_y\mathbf{k}}\rangle + \cos\theta\,|u_{p_z\mathbf{k}}\rangle\right]$$
$$+(\partial_{\mathbf{k}}\phi)\cos\theta(-\sin\phi\,|u_{p_x\mathbf{k}}\rangle + \cos\phi\,|u_{p_y\mathbf{k}}\rangle) \tag{S12}$$
$$= -(\partial_{\mathbf{k}}\theta)\,|u_{p_r\mathbf{k}}\rangle + (\partial_{\mathbf{k}}\phi)\cos\theta\,|u_{p_\phi\mathbf{k}}\rangle, \tag{S13}$$

and

$$|\partial_{\mathbf{k}} u_{p_\phi\mathbf{k}}\rangle = -(\partial_{\mathbf{k}}\phi)(\cos\phi\,|u_{p_x\mathbf{k}}\rangle + (\sin\phi\,|u_{p_y\mathbf{k}}\rangle) \tag{S14a}$$
$$= -(\partial_{\mathbf{k}}\phi)(\sin\theta\,|u_{p_r\mathbf{k}}\rangle + \cos\theta\,|u_{p_\theta\mathbf{k}}\rangle). \tag{S14b}$$

Therefore,

$$\langle \partial_{k_x} u_{p_\theta\mathbf{k}} | \partial_{k_y} u_{p_\phi\mathbf{k}}\rangle = (\partial_{k_x}\theta)(\partial_{k_y}\phi)\sin\theta, \tag{S15a}$$
$$\langle \partial_{k_x} u_{p_\phi\mathbf{k}} | \partial_{k_y} u_{p_\theta\mathbf{k}}\rangle = (\partial_{k_y}\theta)(\partial_{k_x}\phi)\sin\theta, \tag{S15b}$$

and thus,

$$\Omega_z^{p_{\pm1}}(\mathbf{k}) = \mp\sin\theta\left[(\partial_{k_x}\theta)(\partial_{k_y}\phi) - (\partial_{k_y}\theta)(\partial_{k_x}\phi)\right] \tag{S16a}$$
$$= \mp\frac{k_z}{(k_x^2 + k_y^2 + k_z^2)^{3/2}}. \tag{S16b}$$

In Eq. (S16b), the angle derivatives can be easily obtained from the definition [Eq. (S4)].

Finally, by using the rotational invariance, the Berry curvature field for the state $u_{p_m\mathbf{k}}$ is given by

$$\mathbf{\Omega}^{p_m}(\mathbf{k}) = \mp m\frac{\hat{\mathbf{k}}}{|\mathbf{k}|^2}. \tag{S17}$$

Physically, this means that an electron in $u_{p_m\mathbf{k}}$ state experiences a $\mathbf{k}$-space magnetic field generated by the magnetic monopole with the charge $2m$ at $\mathbf{k} = 0$. Equation (S17) can also be cast into a form,

$$\mathbf{\Omega}^{p_m}(\mathbf{k}) = \mp\frac{\langle u_{p_m\mathbf{k}}|\,\mathbf{L}\,|u_{p_m\mathbf{k}}\rangle}{\hbar|\mathbf{k}|^2}. \tag{S18}$$

This explicitly shows that the Berry curvature is directly proportional to the OAM texture.

By integrating the flux over a closed surface containing $\mathbf{k} = 0$, the Chern number can be obtained,

$$C^{p_m} = -\frac{1}{2\pi}\int d\mathbf{S}\cdot\mathbf{\Omega}^{p_m}(\mathbf{k}) = 2m. \tag{S19}$$

This proves that the spinless multifold chiral fermion carries the Chern number $C^{p_m} = -2, 0, +2$. As the spin degree of freedom is included, it splits into two spin up and down pairs for each, so the crossing can effectively be considred as carrying the Chern number $C^{p_m} = -4, 0, +4$.

### 2.3. Orbital moment due to Berry phase

Apart from the atomic OAM $\mathbf{L}$, self-rotation of a wave packet can give rise to orbital moment. According to the Berry phase theory, this contribution is given by

$$\mathbf{m}^{p_m}(\mathbf{k}) = \frac{e}{2\hbar}\mathrm{Im}\left[\langle \partial_{\mathbf{k}} u_{p_m\mathbf{k}}| \times \{\mathcal{H}_{\mathbf{k}} - \mathcal{E}_{p_m\mathbf{k}}\} |\partial_{\mathbf{k}} u_{p_m\mathbf{k}}\rangle\right]. \tag{S20}$$

Here, we derive the orbital moment due to Berry phase for the $l_{\text{eff}} = 1$ multifold chiral fermion model [Eq. (S1)].

For $p_{\pm 1}$ states [Eqs. (S5b) and (S5c)], their derivatives with respect to $\mathbf{k}$ can be easily obtained by using Eqs. (S13) and (S14b), which lead to

$$|\partial_{\mathbf{k}} u_{p_{\pm 1}\mathbf{k}}\rangle = -\frac{1}{\sqrt{2}}\left(\partial_{\mathbf{k}}\theta \pm i\partial_{\mathbf{k}}\phi \sin\theta\right)|u_{p_0\mathbf{k}}\rangle - i\partial_{\mathbf{k}}\phi\cos\theta\,|u_{p_{\pm 1}\mathbf{k}}\rangle\,, \tag{S21}$$

and

$$\mathrm{Im}\left[\langle \partial_{k_x} u_{p_{\pm 1}\mathbf{k}}| \left\{\mathcal{H}_{\mathbf{k}} - \mathcal{E}_{p_{\pm 1}\mathbf{k}}\right\} |\partial_{k_y} u_{p_{\pm 1}\mathbf{k}}\rangle\right] = \mp\frac{1}{2}\left(\mathcal{E}_{p_{\pm 1}\mathbf{k}} - \mathcal{E}_{p_0\mathbf{k}}\right)\sin\theta\left[\left(\partial_{k_x}\theta\right)\left(\partial_{k_y}\phi\right) - \left(\partial_{k_y}\theta\right)\left(\partial_{k_x}\phi\right)\right] \tag{S22a}$$

$$= -\frac{1}{2}\chi v_{\text{F}}\hbar\frac{k_z}{(k_x^2+k_y^2+k_z^2)}, \tag{S22b}$$

where we have used Eqs. (S8) and (S16b) in the second line. Therefore, the orbital moment for $p_{\pm 1}$ states becomes

$$\mathbf{m}^{p_{\pm 1}}(\mathbf{k}) = -\frac{1}{2}e\chi v_{\text{F}}\frac{\hat{\mathbf{k}}}{|\mathbf{k}|^2}. \tag{S23}$$

Meanwhile, for calculating the orbital moment for $p_0$ state, we first evaluate its $\mathbf{k}$-derivative:

$$|\partial_{\mathbf{k}} u_{p_0\mathbf{k}}\rangle = |\partial_{\mathbf{k}} u_{p_r\mathbf{k}}\rangle \tag{S24a}$$

$$= \partial_{\mathbf{k}}\theta\,|u_{p_\theta\mathbf{k}}\rangle + \partial_{\mathbf{k}}\phi\sin\theta\,|u_{p_\phi\mathbf{k}}\rangle \tag{S24b}$$

$$= \frac{1}{\sqrt{2}}\left(\partial_{\mathbf{k}}\theta - i\partial_{\mathbf{k}}\phi\sin\theta\right)|u_{p_{+1}\mathbf{k}}\rangle + \frac{1}{\sqrt{2}}\left(\partial_{\mathbf{k}}\theta + i\partial_{\mathbf{k}}\phi\sin\theta\right)|u_{p_{-1}\mathbf{k}}\rangle\,. \tag{S24c}$$

Therefore, together with Eqs. (S8) and (S16b), we obtain

$$\mathrm{Im}\left[\langle \partial_{k_x} u_{p_0\mathbf{k}}| \left\{\mathcal{H}_{\mathbf{k}} - \mathcal{E}_{p_0\mathbf{k}}\right\} |\partial_{k_y} u_{p_0\mathbf{k}}\rangle\right] = -\frac{1}{2}\left(\mathcal{E}_{p_{+1}\mathbf{k}} - \mathcal{E}_{p_{-1}\mathbf{k}}\right)\sin\theta\left[\left(\partial_{k_x}\theta\right)\left(\partial_{k_y}\phi\right) - \left(\partial_{k_y}\theta\right)\left(\partial_{k_x}\phi\right)\right] \tag{S25a}$$

$$= -\chi v_{\text{F}}\hbar\frac{k_z}{(k_x^2+k_y^2+k_z^2)}, \tag{S25b}$$

and thus

$$\mathbf{m}^{p_-}(\mathbf{k}) = -e\chi v_{\text{F}}\frac{\hat{\mathbf{k}}}{|\mathbf{k}|^2}. \tag{S26}$$

### 3. Photon energy dependence of helicoid topological Fermi arcs

Figure S7**b** shows the photon energy dependence of the circular dichroism (CD). The spherical sections measured at the photon energies at 78 eV, 68 eV, 56 eV, 44 eV, 36 eV cover $k_z$ sections over one complete BZ as shown in Fig. S7**c**. We found that the sign of the CD does not change rapidly with photon energies in this region of the BZ. For instance, around $\overline{\mathrm{M}}$ at $(+0.7, +0.7)$ Å$^{-1}$, the CD always exhibits negative sign independent of photon energies. When crossing the $\overline{\mathrm{M}} - \overline{\mathrm{Y}}$ line from $\overline{\Gamma}$ to $\overline{\mathrm{M}}$ at $(+0.7, +0.7)$ Å$^{-1}$, a CD sign change (or CD intensity modulation) is observed, which is consistent with the calculated $L_y$. This confirms that the observed CD signal can be interpreted as a property of the chiral fermion states. Intensity difference observed as a function of photon energy arise due to different spectral contributions of bulk and surface states, as illustrated in the respective $k_z$ sections in Fig. S7**c**. Note that the section measured at $h\nu$=36 eV cuts through an adjacent BZ, and shows a low photoemission cross section of the Fermi arc surface state.

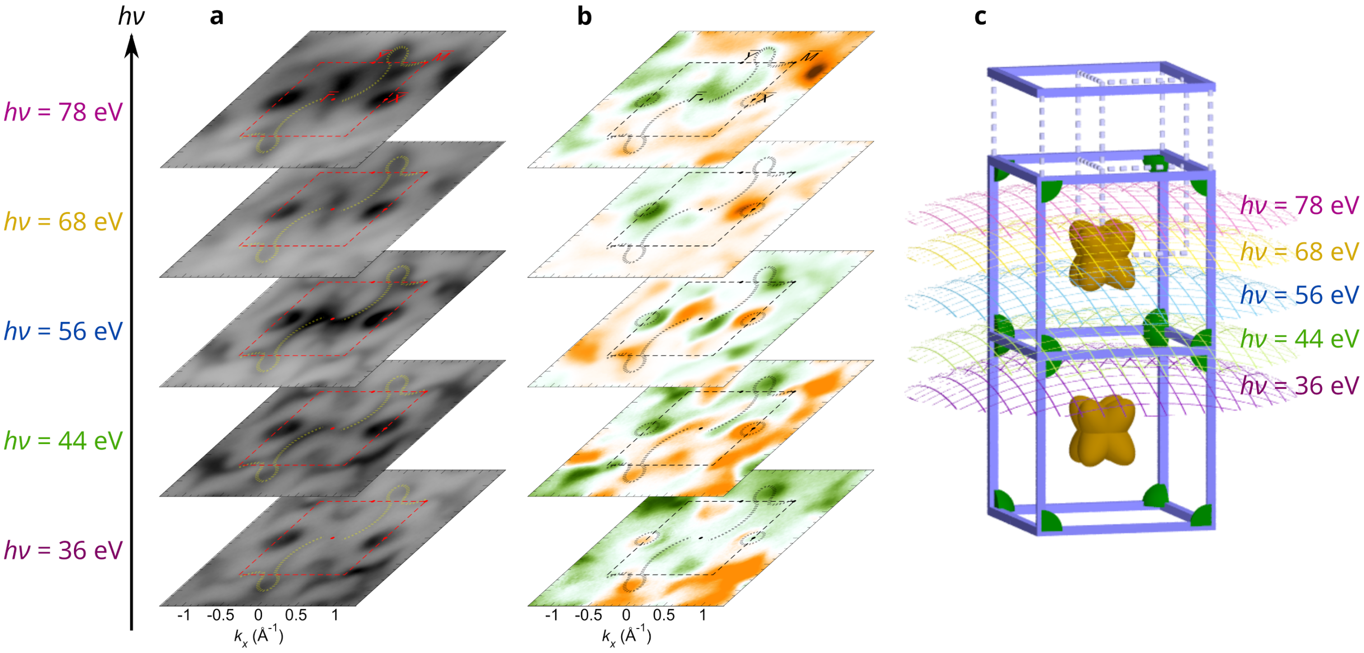


FIG. S7. **Photon energy dependence of helicoid topological Fermi arcs. a** The sum of intensities of photoemission momentum maps measured by LCP and RCP light at the Fermi energy for enantiomer A. The dashed lines, serving as a guide to the eye, indicating the surface Fermi arcs, which are derived from the total intensity maps and remains unchanged across different photon energies, confirming their surface state origin. **b,** The corresponding measured CD maps. **c,** Schematic Fermi surface of CoSi in the bulk BZ with corresponding spherical sections measured at different photon energies.

## 4. Orbital character of the bands in CoSi

Figure S8 shows the orbital characters of the bands in CoSi, for $d_{z^2}$, $d_{x^2-y^2}$, $d_{xy}$, $d_{yz}$, $d_{zx}$ orbitals of the Co atoms (**a**-**e**) and for $p_x$, $p_y$, $p_z$ orbitals of the Si atoms (**f**-**h**). They clearly show that the multifold chiral fermions at $\Gamma$ and $\mathrm{R}$ originate from Co $d$ orbitals, and the weight of Si $p$ orbitals is much smaller.

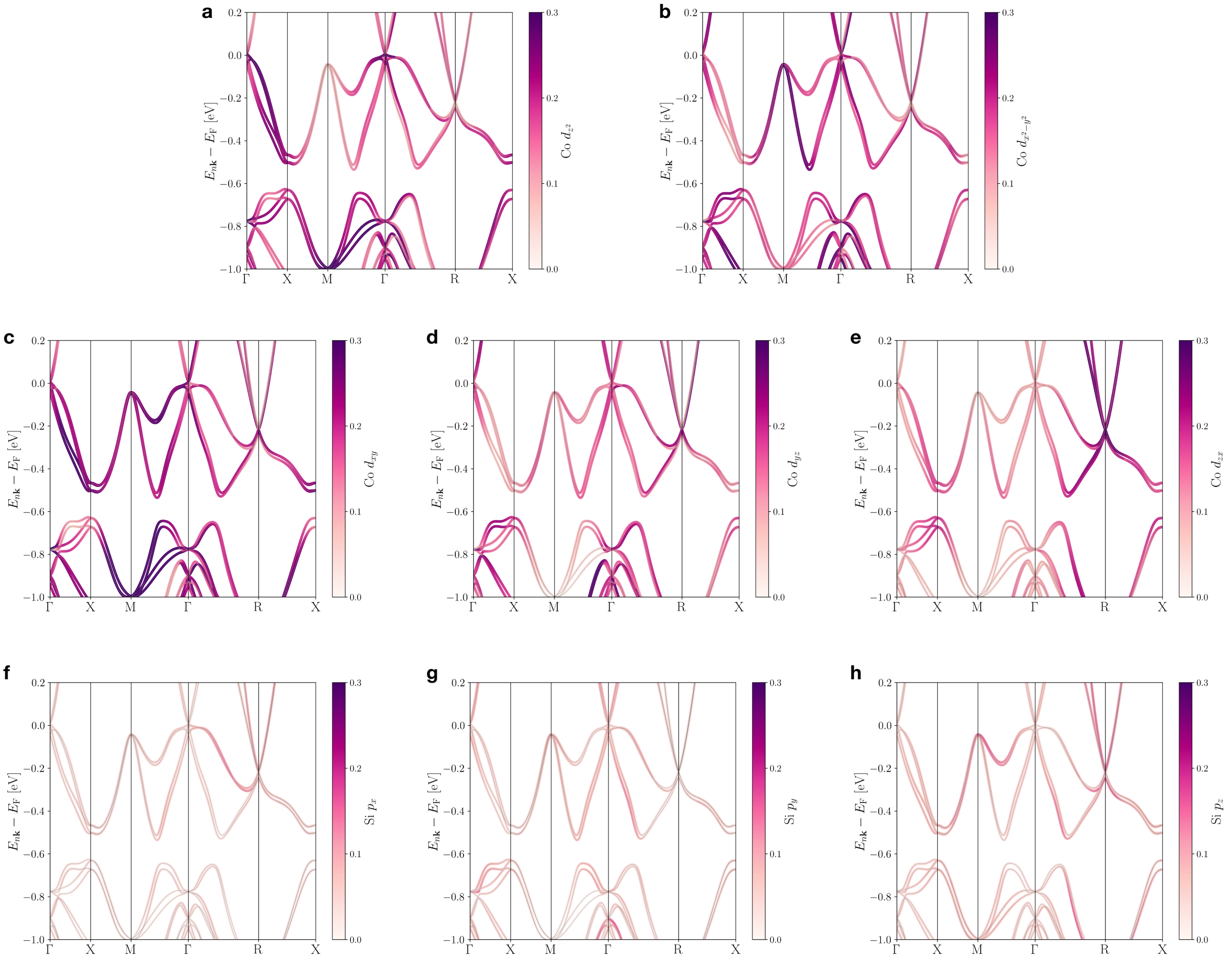


FIG. S8. **Orbital character of the bands in CoSi.** Colorbars represent the weight of orbital characters, where the unit is defined such that '0' and '1' represent zero and full weights, respectively.
